# Reflections on the past, present and future of developmental biology

**DOI:** 10.1016/j.ydbio.2022.05.001

**Published:** 2022-08

**Authors:** Claudio D. Stern

**Affiliations:** Department of Cell & Developmental Biology, Anatomy Building, University College London, Gower Street, London, WC1E 6BT, UK

## Abstract

Developmental Biology embodies some of the most fundamental questions in Biology and can trace its roots back to several thousand years ago; the last 100 years have been particularly extraordinary. In part the advances have been fuelled by new technical advances and knowledge in many other areas, which have contributed to shaping the field as truly interdisciplinary. During those 100 years some of our predecessors identified some key questions and a few important principles especially by trying to find general rules that govern what cells are able to do and how they choose between different options, as well as principles of experimental design that can be used to uncover those rules even before we know their physicochemical underpinnings. But the field has been changing rapidly in the last two decades. Here I present a brief overview of some of the changes that have taken place over the last Century and a personal view of current directions. The picture that emerges is of some dark clouds on the horizon, so this is also a call to arms for our colleagues to try to regain what the field has been losing.

## Highlights

• Developmental biology is in crisis: scholarship and rigour are being eroded.
• Universities are stopping the teaching of classical developmental biology including key concepts.
• Some new technologies encourage big data generation at the expense of uncovering principles.
• These and other conditions are generating a “perfect storm” and threatening the foundations of the field.
• Despite frequent statement of the opposite, most fundamental questions have not yet been answered.

## Graphical abstract

**Figure:**
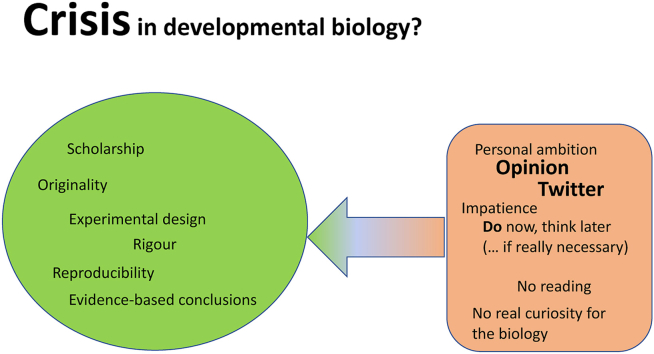

## 1 Introduction

I was extremely fortunate to be an undergraduate and postgraduate student in the 1970s at the University of Sussex, England. This time was special – full of excitement with developmental biology, evolution, neurobiology and genetics and the emerging science of molecular biology. It was almost impossible to avoid being drawn in. I was also lucky to be able to publish papers over 6 decades, to have had exceptional colleagues in 4 universities over this time and to have listened to lectures by some of the true legends of this enormously rich field. I am prompted to write this short retrospective for the volume entitled “The impact of developmental Biology in the last 100 years” partly to reflect on our wonderful history and how the field has evolved over that time, but also to express concern for the current directions and changing perceptions, which I feel could seriously threaten the future of what should remain as a thriving field of enquiry into some of the most fundamental questions in the whole of Biology.

## 2 *Unde venimus* (from where are we coming?): observation, histology, “experimental embryology”, the molecular era

According to the wonderful book “*A history of Embryology”* by Joseph Needham (1959), the systematic study of embryos and other developing systems including plants can trace its origin to at least as far back as the ancient Egyptians and Greeks, including Aristotle. From these early times, a fundamental question was whether at early stages the “germ” is a miniature pre-formed version of the adult that simply grows over time, or whether it increases in complexity, with different parts arising and developing in a set sequence. It seems remarkable that this debate continued for so long, with its peak perhaps in the XVII to the early XIX century as the famous confrontations between the doctrines of Preformation and Epigenesis. Until the mid-XIX century, the study of developing systems was dominated by simple observation (and a few simple experimental approaches including the study of regeneration) aimed at describing the sequence of events and classifying the changing structure of the embryos. From the early 1800s the introduction of chemical staining and histological techniques greatly enriched the study of comparative anatomy of embryos which contributed to generate a very rich body of knowledge on the changes in structure of different developing species over time. At the same time, increasing interest in Evolution (some of its questions mirroring “Preformation and Epigenesis”, often in a religious context) from the late 1700s (Cuvier, Buffon, Geoffroy-St Hilaire, later Darwin and others) brought comparative Anatomy and Embryology to the fore, asking questions about whether evolution could work by altering the developmental process. The emerging questions shifted from “what” happens during development to “how” – what are the key mechanisms responsible for making cells behave appropriately to generate a functional adult organism.

This opened the door for two main disciplines to start contributing: Genetics (especially from the work of Thomas Hunt Morgan and his followers) and Experimental Embryology (arising mainly from the pioneering work of Wilhelm Roux). Genetics uncovered how “mutations”, heritable changes in the germ line, could affect development and body form and, through early attempts at mapping (notably by Morgan's student Alfred Sturtevant), the notion that some of the causative genes were present in some physical sequence in the genetic material whose nature was at that time still unknown. Wilhelm Roux noted that the surviving cell in two-cell-stage embryos in which the other cell had been killed (in his own experiments) behaved differently from the equivalent cell in embryos where the other cell had been carefully removed (in contemporary experiments by Hans Driesch and Hans Spemann): in the first case it formed a half-embryo whereas in the second case it was able to “regulate”, giving rise to a complete but miniature embryo. Roux's reflections on these experiments first pointed clearly to the principle that experimental approaches could be used to explore what cells **can** do and to compare this to what cells **do** do in normal development, and called this approach *Entwicklungsmechanik* (now loosely translated as Experimental Embryology).

In my view, what we have come to know as “Developmental Biology”, the study of the **causative mechanisms** responsible for how organisms develop, arose directly from the juxtaposition of Comparative developmental Anatomy (including Histology) with Genetics and Experimental Embryology, enriched a little later (from the 1920s) by “Chemical Embryology” (from the work of the Needhams, Brachet and others complemented later by the use of antibodies), “Cell Biology” (studying cell behaviours such as cell movements, contacts and adhesions, formalized especially by the work of Trinkaus (1969)) and from the 1970s by the techniques of “Molecular Biology” (including recombinant DNA technologies) [excellent historical accounts of the field after the end of the period covered in Needham's book include Scott Gilbert's “conceptual history” (Gilbert, 1991), Jane Oppenheimer's “essays in the history of embryology” (Oppenheimer, 1967) and Leon Browder's multi-volume “comprehensive synthesis” (Browder, 1985, 1986)]. The application of these approaches in combination were hugely empowering. When “Experimental Embryology” is used judiciously to design pointed experiments, it has the power to uncover the **rules** that govern what cells are able to do at a specific time and place in development and that channel them to a particular outcome from the range of possibilities. Coupled to increasingly sophisticated experimental tools and approaches allows more objective assessment of the outcomes of the experiments, but the logic of experimental design remains central to the discovery of developmental mechanisms, by rigorous testing of different alternative explanations of the developmental events.

The quest for uncovering **rules** even before the physical nature of the driving forces is known probably derives mainly from the work of Hans Spemann and C.H. Waddington and their colleagues in the first half of the XX century. Waddington pioneered clear, almost mathematical definitions of cell properties and events such as fate, induction, competence, determination/commitment, lineage, etc. (Waddington, 1956). This was further enriched after the introduction of the anuran amphibian *Xenopus laevis* as an experimental organism by Pieter Nieuwkoop in the 1950s, which helped to formalize these definitions and introducing a few other concepts, such as *specification* (notably by Jonathan Slack in the first edition of his “*From egg to embryo”* (Slack, 1983) and some of these later refined (Stern, 2004)). This is the “developmental biology” to which I was exposed as a student – it was enormously exciting. The driving force was to deploy pointed experimental design to ask clear questions about the rules, and then whenever possible the physico-chemical nature of the components, responsible for selecting from among a range of possible behaviours of cells as they develop so as to generate a functional organism. As molecular biology and genetics became more sophisticated and were increasingly introduced into the arsenal, combined with experimental embryology, cell tracing and improving microscopy techniques, the 1980s and 1990s then generated a vast wealth of knowledge and wisdom about how cells in embryos can make decisions as they develop, as well as principles of ageing and regeneration, cancer and other areas that can be viewed as aspects of developmental biology.

## 3 “-omics” and data-obsessed biology

Many of the advances over the period discussed above were driven by an expanding array of increasingly sophisticated techniques and approaches, which could be deployed to ask the fundamental questions increasing precision and clarity. I have argued elsewhere (Bizzarri et al., 2019; Stern, 2019) that around the turn of the current Century, the explosion of “-omics” techniques (high throughput data acquisition) may have been responsible for the start of a serious erosion especially in the importance placed on experimental design to ask clear questions where the experiment attempts to reduce the number of variables to just one, which represents the “core” of the question. High-throughput data acquisition, together with better computational methods generated a “data-driven” culture in biology where it is the computer that is charged to finding connections that might represent answers to questions that have not yet been asked. This is obviously very powerful, but in my view, it is only powerful when coupled with a desire by the experimenters to increase their wisdom, or understanding, of the causative forces responsible for biological processes, rather than just increasing numbers of associations. The casualty is not just experimental design, but the whole Waddingtonian ethos of uncovering **rules** that govern the behaviour of cells, tissues and systems. In the last 20 years the community has become obsessed with massive data collecting and semi-automated (computational and statistical) methods of “self-organization” of the data generated, but with little integration to attempt to understand mechanisms. In the remainder of this piece I suggest that alongside this, several other driving forces have contributed further to the erosion of “Developmental Biology” as such.

## 4 Fashions

Over my career I have witnessed several passing fashions. Some of them were driven by new technologies, but not all. As an undergraduate in the early 1970s cyclic-AMP was all the rage, not only for what it was being found to do in Dictyostelium but as a quite general messenger and signal with many functions. Serotonin also had a brief similar period of notoriety as a potentially powerful signal. Later that decade, monoclonal antibodies came in, offering wonderfully specific ways to explore the distribution of particular components in cells, tissues and embryos. Along with this, the Extracellular Matrix became a subject of intense interest, along with studies on cell-cell and cell-substrate adhesion and their relations to cell motility (mostly in vitro), which also led to exploration of mechanical forces that might contribute to shape tissues by modulating such adhesions (Browder, 1986; Trinkaus, 1969). As recombinant DNA techniques evolved in the 1980s, it became possible to localize gene expression by in situ localization of specific mRNAs (initially by laborious and rather messy radioactive in situ hybridization, but soon radically transformed by the introduction of non-radioactive methods, using digoxigenin-labelled probes which are still in use today), and quantification of gene expression (at that time mainly relying on RNase protection assays, again with high levels of radioactivity being handled). These methods greatly enriched the “developmental anatomy” by painting embryos with the locations where many genes are expressed at different stages. Confocal microscopy started to influence how embryos and tissues could be imaged, from the early 1980s.

At this time, systematic forward genetics especially in two key “genetic” organisms, Drosophila and the nematode C. elegans, started to highlight key genes, including transcription factors, with “essential” roles in development – the most notable example of this, coupled with a transformative classification of the genes to define developmental “epochs”, is the Nüsslein-Volhard and Wieschaus classic (Kimble and Nusslein-Volhard, 2022; Nüsslein-Volhard and Wieschaus, 1980). It was not long before the next major landmark in 1984, the discovery, mainly by Walter Gehring, of a highly conserved DNA sequence motif he called the Homeobox, associated with transcription factors that play crucial and universal roles in cell identity and encoding axial position in the embryo. In the mid-1990s the first genes involved in setting up left-right asymmetry were discovered, which also opened up a rapidly growing field of research. The two or three decades that followed were marked largely by vast numbers of papers trying to assign specific developmental functions to particular genes, sometimes formulaically entitled something like “Gene XXX is required for YYY process”. Facilitating this, in the late 1980s and early 1990s, embryonic stem (ES) cells were generated for the first time, opening the door for much more efficient gene targeting in mouse (two decades later this was made even more efficient by the introduction of CRISPR-Cas9 technology, making it much easier to target genes in almost any organism) (Capecchi, 2022). Of course ES cells themselves (and the later-available iPS cells that can be generated from many if not all somatic cell types, as a result of the pioneering work by Shinya Yamanaka and colleagues) (Takahashi and Yamanaka, 2006), and the production of “organoids” and even “embryoids” from such cells later entered the scene as developmental systems in their own right.

There are a few current trends and fashions, the most salient ones probably being “mechanobiology” (or the “physics of living systems”), computational modelling of different types, single-cell approaches including RNA-sequencing (and increasingly “multi-omics” applied to single cells), the generation of “embryoids” and “organoids” (and studying them by single-cell and mechanobiological approaches to them). It is perhaps curious that some of the topics that were once fashionable but then died down later made a comeback as if they were completely new. An example of a current fad is “metabolism” as a driver of developmental complexity – this was first proposed a Century ago by Charles Manning Child, 1914, 1941; recent “metabolomics” and a better array of chemical inhibitors and agonists have driven a return to this view, although the types of processes that are likely to be orchestrated primarily by such mechanisms are not always clear, apart from some fairly obvious events such as the pattern of vascularization that is dependent on spatiotemporal variations in oxygen demand and consumption.

There is of course nothing wrong with “fashions”, and they will occur naturally. But what I find particularly concerning is that so many colleagues, funders and journals seem to choose what they work on and how based on what they perceive to be trendy at the time. History should have demonstrated that this is a recipe for disaster for today's early career researchers: a topic that is fashionable now is likely to be replaced by a different one tomorrow (until of course it all repeats itself in the more distant future … !). Over the last few years, I have been shocked by seminars, or grant applications, that start with a statement like: “I am interested in how cells make decisions”, then followed by one or more of the trendy approaches but with little explanation as to how this will answer the initial question. Again, as with “omics”, the problem seems to be a broken connection between asking a tractable question and designing a clear experiment that will answer it, unambiguously. This requires reducing the variables to a minimum, ideally just one. It seems that our discipline, despite its long tradition of “Experimental embryology”, is losing the ability to do this, but more frighteningly, perhaps the curiosity to discover “how” has become less of a burning driving force.

## 5 “Model” systems

The “model systems” also changed over time. In the early 1970s I recall travelling to the Royal Society with my classmates to listen to a lecture by Sydney Brenner where he introduced a new organism, the nematode *Caenorhabditis*, which has so few cells and a small genome that he made the prediction that once we knew all the lineage relationships between the cells, and the effects of interfering with the expression of all genes (one by one), it should be possible to understand all of development (see also Kimble and Nusslein-Volhard, 2022). We argued whether he might be right on our trip back to University, and for some time thereafter, and the general feeling was that this was highly unlikely – of course we were proved correct. But it is ironic in retrospect that it was the same Sydney Brenner who later (around 2010) cynically stated that Biology was being dominated by “*low input*, high throughput, no *output*” research – the idea that simply listing all lineage relationships and effects of all knockdowns could generate wisdom might be considered an example of the same view! Nevertheless *C. elegans* did turn out to be a very useful model system that led to many important general discoveries. Another new model that emerged over the 1980s–1990s was the zebrafish *Danio rerio*, which curiously attracted many scientists who had previously been working with *Drosophila* as a genetic system – the expectation from these was at that time mainly that it should be possible to generate mutations in every gene and examine their phenotypes, and this would be helped by having a very transparent organism excellent for live imaging, which parallels the reasons why Brenner chose the nematode.

As with fashionable topics, there is nothing wrong with new organisms being exploited. But there has been a growing trend to favour a smaller number of organisms and looking for “conserved mechanisms”, ignoring all that does not appear to be “conserved”. The history of Developmental Biology does amply justify this principle, but also strongly justifies the opposite view. Decades of work on *Drosophila* genetics led to the discovery of probably the vast majority of developmentally (and more broadly) important genes. In fact, the pathways in which these genes act are themselves strongly “conserved” among metazoa, but interestingly the specific processes and organ systems in which they play a role are not always the same. Clearly the same toolkit has been deployed multiple times during evolution to achieve different outcomes, and it appears that the tools (pathways) themselves are under stronger selection than the processes they control. Is it therefore justifiable only to study mechanisms and processes deemed to be “conserved”? The rich literature of Developmental Biology has many examples illustrating just the opposite. Key principles may emerge only by studying the differences between different organisms, especially when they use different mechanisms to achieve apparently the same end. One example in development is how the main embryonic body/gut axis (between the site of gastrulation and the opposite end) is specified in different animals depending on the time of transition between maternal and zygotic gene expression: in animals where this transition occurs only after many cell divisions (most invertebrates and anamniote vertebrates), localization of maternal determinants are mainly responsible for this, whereas in animals where the transition occurs early the control involves differential gene expression at an early stage and more stochasticity. Another example, perhaps more dramatic, arises from examining the different mechanisms by which organisms gastrulate (generate the endoderm and associated mesoderm). Even in apparently similar species, such as in Ctenophores and Cnidarians, seemingly closely-related animals appear to use quite different cell behaviours to generate very similar patterns (e.g. Byrum and Martindale, 2004; Martin-Duran et al., 2016) – these observations led to the view that protostomy and deuterostomy may have arisen independently several times in evolution, but more generally, that the outcome of a developmental process (like the “gastrula” pattern) may be under stronger selective pressure than the mechanisms by which it is generated.

A second reason for not limiting ourselves to a few models is that each experimental system offers different advantages and disadvantages for experimental designs. Some are particularly suitable for forward genetics (screens), others for targeted (reverse) genetics, others to study cell behaviours in vivo, others for challenging cell interactions by transplantation or other manipulations at different stages. The questions that can be asked in each case may be more suitable to one or another organism. Therefore, counterintuitively, by restricting our studies to just a small number of “model” organisms, we may risk missing many of the important principles.

## 6 Prejudices

### 6.1 Funding decisions and changing perceptions on “research impact”

The discussion about “model” organisms has particular bearing on funding bodies which are often concerned with delivering what they view as “impact”: the applicability of research findings. Many of the funding bodies are primarily interested in human health so they tend to favour research on organisms that appear to be closer to humans. I do feel that this is misguided; one reason, related to the considerations outlined in the previous section, is that the most commonly used models for this (e.g. mice) may execute some developmental processes much more differently to human embryos than other animals that appear to be less closely related. An example is how rodents and non-rodents (including primates, other groups of mammals and avian embryos) generate the primitive streak (see Stern and Downs, 2012). Another, even more important, reason is that the application of this principle to decide what should be funded or not risks failing to discover the most important principles and rules. History has provided many examples where findings have led to very unexpected, yet hugely important discoveries with direct relevance to human health and wellbeing. A well-known example is the discovery of Penicillin, of course, but probably most fundamental knowledge derives from the combination of diverse experimental systems and approaches that have been used over time. In my view, a good question coupled by well designed experiments can generate much deeper and fundamental knowledge than restricting oneself to those experiments that can be done in a pre-selected organism just because it is perceived to be “closer” to humans.

### 6.2 Journal editors and reviewers

Prejudices like those above also apply to journals and what they deem “interesting” and publishable. The often-called “top” journals, which are run by full-time professional career editors, have over the last few decades tended to use journalistic (tabloid) criteria to pre-judge the level of what they perceive to be the “general interest” of a paper. This tends to select papers that deliver a very simple story and drives authors to feel that “spin” is necessary to publish in those journals. This was not always the case. Once the editors of these journals did look for papers that move the field forward, and particularly favoured papers that offered an **elegant** demonstration of the findings and conclusions (through beautiful experimental design). Now, current fashions seem to dominate the choice of what is “publishable” – just looking at the huge number of “Atlas”-type papers reporting transcriptional diversity (with a number of different interpretations about the significance of such diversity) based on single-cell-RNA-sequencing (scRNAseq) data is frightening. Most of these papers are not ground-breaking (other than technically, sometimes), they rarely uncover new principles, and most have little or no experimental design – just many data and speculations.

Reviewers are also increasingly influenced by such criteria, even in journals that are run by scientific colleagues. Many recent reviews I have seen include attitudes like “it would also be nice to have much more information about XXX” but without regard to whether this information would further illuminate the question being asked, or in what way. Given that what would be “nice” is so subjective, this should not be part of the review process. It seems that we are in desperate need of better training for reviewers and editors.

### 6.3 Technologies and questions

Some funders and publishers are taking decisions about what is “interesting” or “important” based on how “modern” the technology used is perceived to be, irrespectively of how well the chosen methods can answer the questions. But a view which I have heard expressed increasingly is that the field of developmental biology has been so successful that it has already provided answers to all the most important questions (apart from details), and what remains is only the application of this knowledge to more “useful” ends. For anyone who thinks the latter is the case, I would strongly recommend going back to read Waddington's 1956 book “Principles of embryology” (Waddington, 1956) where he so clearly outlines the key principles and questions that were open at that time (and evaluates a range of possible answers to them) – it is inescapable that we are still just as ignorant of the answers to most of the fundamental questions as he was. If anything, we have filled in many details, but most of the really big questions, and especially the most interesting ones, remain unanswered.

## 7 University syllabuses and the gradual erosion of developmental biology courses

Over the last two decades or so, many university degree programmes have stopped delivering courses with the title “Developmental Biology” (and “Embryology” in medical curricula). In some cases this has been done because of a perception that this whole discipline is not as attractive to students as say, “Stem cells and regenerative biology”, or similar titles, which are seen again to reflect fashions and trends. The casualty in this case is that very few students are currently exposed to the very core of Developmental Biology – the very **rules** that have defined this discipline for the last 120 years, and to the value of experimental design. Students are increasingly unable to design experiments that aim to establish direct causality. From the perspective of my exposure to the changes in university programmes in the last few decades, I think that there have been two main driving forces: one is yet again the rise in data-collection type of approaches (the “omics” argument), but the other is that in the earlier training years, students are mainly asked to learn “facts”, which represent the conclusions of previous work, rather than how these conclusions were reached and how. So many students are not trained in formal epistemology or the difference between objective findings and subjective/interpretative conclusions, or how to combine the findings and conclusions from different studies in scientifically rigorous ways.

## 8 Less reading

**Fig. 1:**
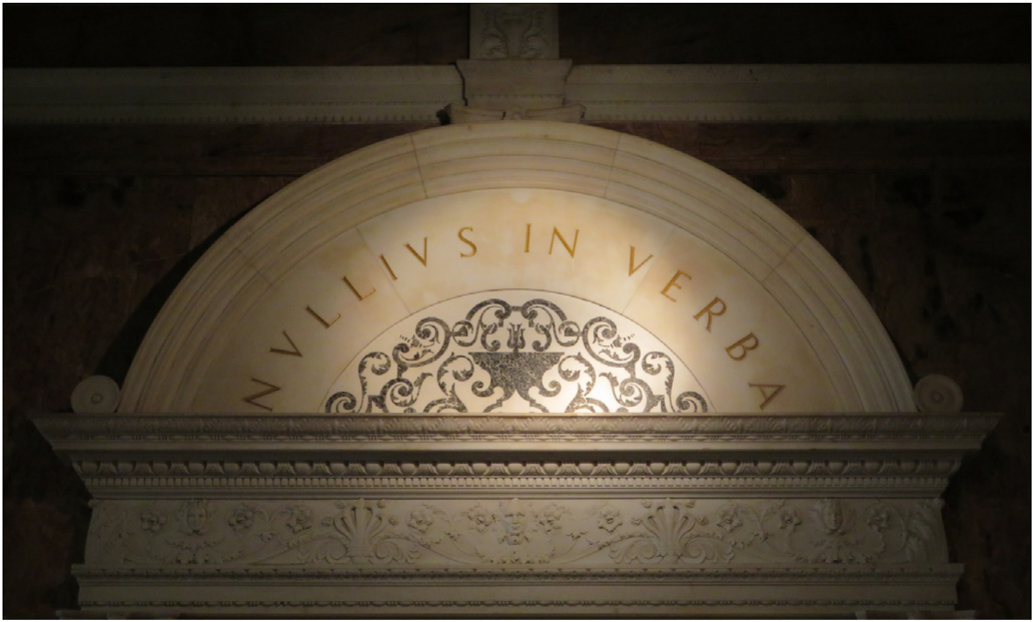
Inscription above an entrance in the Marble Hall of the Royal Society in London, with the motto of the society: “*Nullius in verba*”. Picture by Ellen Embleton, courtesy of the Royal Society.

Partly for the same reasons as those that shape university courses and how students are trained, with more emphasis on conclusions than on the original findings, it seems that increasingly, papers are cited on the basis of their title (at most their abstract) or even on the perception of what a particular author *might* have said about a particular topic, irrespective of the contents of the paper. From its foundation in 1660, the Royal Society has chosen as a motto “Nullius in Verba” (Fig. 1) – this is a quotation from a Latin translation of Horace abbreviated from “*Nullius addictus jurare in verba magistri*”, which was translated by Andrew Huxley as “not committed to swearing by the words of any master” (interpreted as “rejection of authority as a source of truth). A more modern rendering might be “take no-one's word for it”.

Of course, we still have some extremely scholarly colleagues who are deep thinkers, but it seems that the attitude caricatured above is becoming very widespread, and also influences the decisions made by editors and reviewers and by grant funding panels. As an undergraduate, we spent a lot time discussing our views of developmental mechanisms with other students. I don't see this happening very much anymore. It appears that something perceived as “work-life balance” and increasing amounts of time spent on social media are eroding the time available for deep and engaging thought and discussion. Perhaps this is at the root of the general lack of motivation and biological curiosity that seems to be increasingly pervasive.

Because of the random way many papers are cited, measures of “impact” based on citations are becoming increasingly flawed. Reviews are cited more often than primary papers (which drives many journals to aim to publish as many of the former as possible), and more recent papers tend to be cited more than those that first made a particular discovery. This system rewards “me-too”, secondary and repetitive work rather than originality, yet the measure of citations (including the “h-index” and similar measures) is widely used. Some countries go even further: they evaluate the importance or value of the research of an individual by adding up the impact factor of the *journals* in which the work was published, and irrespective of the role of the author being evaluated (first/last versus middle of many, reviews versus primary literature, or their role) or other more important features. Moreover, many truly influential papers have taken a long time before their influence became manifest – the best cited example is Mendel's work on genetic inheritance. Other findings that are very influential are hardly cited any more because they were so important that they became “common knowledge” (such as the structure of DNA described by Watson and Crick). I have heard colleagues justify not citing a particular paper that was the first to demonstrate a principle by stating that it was “old”! Perhaps some of this lies at the root of the re-emergence of “trendy” topics after some years.

## 9 A tower of Babel …

The demise of university courses that teach the principles of Developmental Biology (and in turn, increasingly, that people teaching related subjects have not themselves been taught this), along with a decrease in scholarship and a historical perspective of the subject, are contributing to a strange phenomenon. The core language of development, which established the main concepts that can help to formulate clear hypotheses and to design good experiments to compare what cells **can** do (“developmental potential”) with what cells **do** do (“fate”) is now being seriously eroded. Instead, individuals seem to pick their own definitions. An example is “specification” – this is classically defined as the tendency of a cell or group of cells to develop in a specific direction when deprived of its normal cues, especially when cultured in a “neutral” environment (Slack, 1983) – of course this is only feasible in some systems but the definition is still useful in many ways. However, increasingly the word “specification” is used to refer to cells that express some chosen gene(s) that are deemed to represent a state that is transitional on the way to a differentiated cell type. The choice of genes is not always the same, yet the conclusion that cells may be specified in a particular direction can become dominant in a study in the absence of any functional evidence. This is particularly problematic in the case of analysis of scRNAseq datasets where cell diversity is often interpreted as mainly reflecting a developmental succession of states even when the data are obtained from a single embryo, and branch points in cell relatedness are given a label of “specification” or “cell fate choice” just based on the interpretation of expression of one or a few familiar genes. Very few current papers follow up these by experimental tests of lineage relationships. The resulting diversification of the core language of developmental biology represents a veritable Tower of Babel, where colleagues can no longer communicate with each other clearly because of different interpretations of the same words, as well as the choice of different words to express the same situation. This is very dangerous.

## 10 The future

I have expressed a particularly gloomy view of the current directions of the field of Developmental Biology (and others have recently expressed similar views, including Gilbert, 2017; St Johnston, 2015; Wallingford, 2019; Zon, 2019), but I am truly concerned about the combination of these factors which can constitute a “perfect storm”. I hope I am wrong. The field remains one of the most fundamental parts of Biology and it should retain (or regain) the level of excitement that it generated a few decades ago. But I feel that to achieve this we need to reverse some of the current problems as outlined above, especially to return to appreciate what is a “good question” and what constitutes an elegant experimental approach to answer that question. All cells in an organism have the same genetic information yet they generate often huge complexity as they diversify in the appropriate locations at the correct time and generate form and pattern as well as an array of identities, dynamic behaviours and functions. The key quest is to find the “computer program” that contains the instructions to build an organism, and the mechanisms responsible for its evolution over longer periods. This constitutes wisdom rather than large quantities of data, and I feel that it may be difficult to gain such wisdom until we free ourselves from the current data-collecting obsession and return to designing beautiful experiments. For our young (and some not so young) colleagues, I would strongly recommend reading (and re-reading) some of the most inspirational writings of the giants on whose shoulders we still stand, such as Waddington's “Principles of Embryology” (Waddington, 1956) as well as Peter Medawar's equally immortal “Advice to a Young Scientist” (Medawar, 1979).
